# A Case of Puerperal Convulsions, Four Days after Delivery

**Published:** 1890-11

**Authors:** V. O. Lockhart

**Affiliations:** Homer, Ga.


### A CASE OF PUERPERAL CONVULSIONS FOUR DAYS AFTER DELIVERY.

BY DR. V. O. LOCKHART, HOMER, GA.

There is no condition of human suffering which appeals more forcibly to our sympathies, than that of the terrible eclamptic seizure. "The expression is horribly altered; the globes of the eyes are turned up under the eyelids, so as to leave only the white sclerotics visible, and the angles of the mouth are retracted and fixed in a convulsive grin. . . . . Frothy saliva collects about the mouth, and the whole appearance is so changed as to render the patient quite unrecognizable. . . . . The convulsive movements soon attack the muscles of the body; the whole muscular system is thrown into rapidly recurring convulsive spasms." Such is a portion of the graphic description given by Playfair in his admirable work on obstetrics. It has been my fortune to see quite a number of such cases during twenty years of practice, but the case I am now to record was the worst I ever saw, and I report it on that account, and for the additional reason that it occurred four days after delivery—the longest time I ever knew or read of.

It was on the night of July 16th last, and my patient was a mulatto woman, of small size, thirty-five years of age, the mother of several healthy children. I reached her at 4 o'clock A. M., and found her in a violent convulsion. Her previous health had been good, except for a short time previous to her confinement she had suffered from severe headaches, swelling of the feet, indigestion, etc. She had been attended by a midwife in her labor, and her husband stated that it had been easy and natural and was at term. The first convulsion had come on about ten or eleven hours before my arrival, and her husband stated that just before it occurred she complained of a violent headache and blindness. One convulsion had followed another very rapidly and her husband had counted them up to 1 o'clock A. M., at which time she had had twenty. I suppose in all she must have had over thirty. Her pulse was 135 per minute, full and strong, and her temperature 103 degrees in the axilla. She was also suffering from septicæmia, which I immediately recognized by the peculiar odor of that disease. I did not think it possible for her to live more than an hour, but I immediately bled her from the left arm, a full, rapid stream, continuing until she was pulseless or nearly so. The amount of blood taken was nearly two pints. I immediately followed the bleeding by a hypodermic injection of morphine, using nearly one-half grain of the drug, together with 1–60 grain sulph. atropia. To my surprise she did not have another convulsion, except a very light one, about forty-five minutes after the injection. One hour later I succeeded in getting her to swallow twenty grains of bromide potassium. I then gave her a thorough vaginal douche of warm water and carbolic acid, and left her resting easy until 10 o'clock A. M., when I returned and found her still resting. Her temperature had gone down to normal and she had passed water, but was still unconscious, lying in a profound stupor. She could, with difficulty be made to swallow, and I kept up the bromide in twenty grain doses every four hours till next day, when I returned and found her much better but still unconscious. I had given her a cathartic, which had acted well. I had also kept up the carbolized injections. From that time she went on to a good recovery, but she remained unconscious nearly three days from the time the convulsions ceased, and it was nearly a month before her mental capacities were fully restored. This case shows the value of venesection. I think she would have died in a few hours had she not been bled.

